# Clinicopathological Implication of Long Non-Coding RNAs SOX2 Overlapping Transcript and Its Potential Target Gene Network in Various Cancers

**DOI:** 10.3389/fgene.2019.01375

**Published:** 2020-01-23

**Authors:** Yishu Li, Mengyu Du, Shengsheng Wang, Jin Zha, Peijie Lei, Xueqi Wang, Di Wu, Jianhua Zhang, Denggang Chen, Dong Huang, Jing Lu, Heng Li, Min Sun

**Affiliations:** ^1^ Department of General Surgery, Taihe Hospital, Hubei University of Medicine, Shiyan, China; ^2^ Department of Anesthesiology, Institute of Anesthesiology, Taihe Hospital, Hubei University of Medicine, Shiyan, China; ^3^ The First Clinical School, Hubei University of Medicine, Shiyan, China; ^4^ Institute of Medicine and Nursing, Hubei University of Medicine, Shiyan, China; ^5^ Department of Medical Imaging, Taihe Hospital, Hubei University of Medicine, Shiyan, China; ^6^ Hubei Key Laboratory of Embryonic Stem Cell Research, Taihe Hospital, Hubei University of Medicine, Shiyan, China

**Keywords:** SOX2-OT, cancer, prognosis, clinicopathological significance, meta-analysis

## Abstract

**Background:**

SOX2 overlapping transcript (SOX2-OT) produces alternatively spliced long non-coding RNAs (lncRNA). Previous studies of the prognostic role of SOX2-OT expression met with conflicting results. The aim of this study was to properly consider the prognostic role of SOX2-OT expression in several cancers. In addition, the regulative mechanism of SOX2-OT is explored.

**Methods:**

PubMed, EMBASE, and Cochrane Library and The Cancer Genome Atlas (TCGA) database were comprehensively explored to recover pertinent studies. We conducted an extensive inquiry to verify the implication of SOX2-OT expression in cancer patients by conducting a meta-analysis of 13 selected studies. Thirty-two TCGA databases were used to analyze the connection between SOX2-OT expression and both the overall survival (OS) and clinicopathological characteristics of cancer patients using R and STATA 13.0. Trial sequential analysis (TSA) was adopted in order to compute the studies’ power.

**Results:**

Thirteen studies involving 1172 cancer patients and 32 TCGA cancer types involving 9676 cancer patients were eventually selected. Elevated SOX2-OT expression was significantly related to shorter OS (HR = 2.026, 95% CI: 1.691–2.428, *P* < 0.0001) and disease-free survival (DFS) (HR = 2.554, 95% CI: 1.261–5.174, *P* = 0.0092) in cancer patients. Meanwhile, TSA substantiated adequate power to demonstrate the relationship between SOX2-OT expression and OS. The cancer patients with elevated SOX2-OT expression were more likely to have advanced clinical stage (RR = 1.468, 95% CI: 1.106–1.949, *P* = 0.0079), earlier lymphatic metastasis (*P* = 0.0005), earlier distant metastasis (*P* < 0.0001), greater tumor size (*P* < 0.0001), and more extreme tumor invasion (*P* < 0.0001) compared to those with low SOX2-OT expression. Meta-regression and subgroup analysis revealed that follow-up time, sample type, and tumor type could significantly contribute to heterogeneity for survival outcomes. The follow-up time could significantly explain heterogeneity for tumor, node, metastasis (TNM) stage. Furthermore, up to 500 validated target genes were distinguished, and the gene oncology (GO) and Kyoto Encyclopedia of Genes and Genomes (KEGG) analyses demonstrated that the validated targets of SOX2-OT were substantially enriched in cell adhesion, mRNA binding, and mRNA surveillance pathways.

**Conclusions:**

Elevated expression of SOX2-OT predicted a poor OS and DFS. Overexpression of SOX2-OT was correlated with more advanced tumor stage, earlier lymphatic metastasis, earlier distant metastasis, larger tumor size, and deeper tumor invasion. SOX2-OT-mediated cell adhesion, mRNA binding, or mRNA surveillance could be intrinsic mechanisms for invasion and metastasis.

## Introduction

SOX2 overlapping transcript (SOX2-OT) is a long non-coding RNA located in 3q26.33 locus. Its third intron harbors SOX2 gene which encodes the transcription factor SOX2, an established pluripotency state modulator (Avilion et al., 2003; Fong et al., 2008; Han et al., 2018). Several studies revealed that SOX2-OT levels were consistently positively correlated with SOX2 levels. SOX2-OT plays a role in proliferation of cells and SOX2 regulation (Amaral et al., 2009; Hou et al., 2014; Shahryari et al., 2014; Shahryari et al., 2015).

It has been shown that lncRNA SOX2-OT is overexpressed in a number of human cancers as an oncogene promoting tumorigenesis and cancer progression, including ovarian cancer, breast cancer, pancreatic ductal adenocarcinoma, cholangiocarcinoma, hepatocellular carcinoma, esophageal squamous cell carcinoma, osteosarcoma, non-small cell lung cancer, and gastric cancer (Iranpour et al., 2016; Zhang et al., 2016; Zou et al., 2016; Wang et al., 2017a; Wang et al., 2017b; Han et al., 2018; Li et al., 2018a; Li et al., 2018b; Sun et al., 2018; Tian et al., 2018; Wei et al., 2018; Xie et al., 2018). SOX2-OT is co-upregulated with SOX2 and OCT4 in esophageal squamous cell carcinoma and potentially involved in maintaining the pluripotent state of stem cells (Shahryari et al., 2014). Although these articles established the critical role of lncRNA SOX2-OT expression in some cancers, the prognostic value of SOX2-OT expression in numerous other cancers remained uncharacterized (Shahryari et al., 2015; Castro-Oropeza et al., 2018; Farhangian et al., 2018). In addition, inconsistent results were obtained in several studies on the association between SOX2-OT expression and clinical features such as tumor size, clinical stage, and tumor invasion (Shi and Teng, 2015; Zou et al., 2016; Wang et al., 2017a; Li et al., 2018b; Sun et al., 2018).

The evidence above showed that SOX2-OT is involved in tumor progression. Moreover, an earlier meta-analysis study published in 2018 had revealed that the overexpression of SOX2-OT was significantly correlated with the overall survival (OS), clinical stage, lymph node metastasis, distant metastasis, and tumor differentiation of cancers (Song et al., 2018). However, the sample size of the study was restricted, and the relationship between SOX2-OT and other clinicopathological characteristics was not explored (Song et al., 2018). As described below, we have conducted a more comprehensive trial sequential analysis (TSA) on the applicable literature and searched The Cancer Genome Atlas (TCGA) database to study the prognostic value of SOX2-OT in patients with several types of cancer. We additionally explored the potential target genes of SOX2-OT through gene ontology (GO) and Kyoto Encyclopedia of Genes and Genomes (KEGG) analyses, and the potential mechanisms of SOX2-OT in tumor progression are also discussed.

## Methods

### Search Strategy

Studies on the prognostic roles of SOX2-OT in cancer patients that were published as of October 1st, 2019 were extracted from the electronic databases PubMed, EMBASE, and Cochrane Library using the terms (1) “SOX2-OT” OR “NCRNA00043” OR “SOX2OT” OR “SOX2 overlapping transcript” OR “SRY-box transcription factor 2 overlapping transcript” AND (2) “tumor OR cancer OR carcinoma OR neoplasm OR metastasis”. The search strategies are illustrated in **Supplementary Table 1**. The search and selection of articles for the study were conducted as described previously (Sun et al., 2019).

### Inclusion and Exclusion Criteria

Studies entering this analysis met these requirements: (1) definitive diagnosis or histopathological confirmation for patients with cancer; (2) the expression of SOX2-OT must be measured by quantitative real-time polymerase chain reaction (qRT-PCR); (3) the hazard ratios (HRs) and their 95% confidence intervals (CIs) for survival parameters based on SOX2-OT expression levels were promptly available or could be calculated indirectly; and (4) the representative and accurate studies were selected to avoid unnecessary cohort overlapping. Studies that have satisfied the abovementioned inclusion requirements were further ruled out if they had any of the following features: (1) duplicated articles or data; (2) non-human studies; (3) review articles or letters; (4) articles in non-English languages.

### Quality Assessment of Included Studies

The quality of the included studies was assessed using Newcastle-Ottawa Scale (NOS), with scores ≥ 6 considered high quality. A ‘‘star system’’ was applied for case-control studies (**Supplementary Table 2**).

### Data Extraction

The following information was extracted from each study: (1) first author; (2) publication year; (3) nationality, sample size, tumor type, and clinicopathological characteristics of involved patient population; (4) the assay method and cut-off value of SOX2-OT expression levels; (5) HRs of SOX2-OT expression for OS and disease-free survival (DFS). If the HRs for OS and DFS were calculated by both univariate and multivariate analyses, the latter were our first choice for these results and were adjusted for confounding factors. If a study did not report HRs, we estimated HRs and their corresponding 95% CIs using the procedure described by Parmar et al. (1998) and Tierney et al. (2007). The data of Kaplan-Meier curves were regained by Engauge Digitizer software (version 9.8, http://markummitchell.github.io/engauge-digitizer). This process was repeated three times to decrease variability. Discrepancies were resolved through discussion and review of extraction until consensus was reached on a final list of factors targeted by each study.

### Statistical Analysis

All the HRs and their 95% CIs were integrated to evaluate the association between SOX2-OT expression and prognosis. If the pooled HR < 1 and their 95% CI did not overlap the invalid line in the forest plot, the elevated expression of SOX2-OT predicted a good OS. The heterogeneity of the pooled results was examined *via* Cochrane’s Q test and Higgins’ I-squared. If *P* ≥ 0.1 and I^2^ ≤ 25%, we disregarded the influence of heterogeneity and pooled the overall result using a fixed effects model, otherwise employing the random effects model. Potential publication bias was assessed by a funnel plot and Egger’s test (Stuck et al., 1998) conducted using the “metafor” and “meta” packages of R (version 3.2.3). All of the abovementioned methods followed the Meta-analysis of Observational Studies in Epidemiology (MOOSE) Checklist.

## Results

### Identification of Eligible Studies

**Figure 1:**
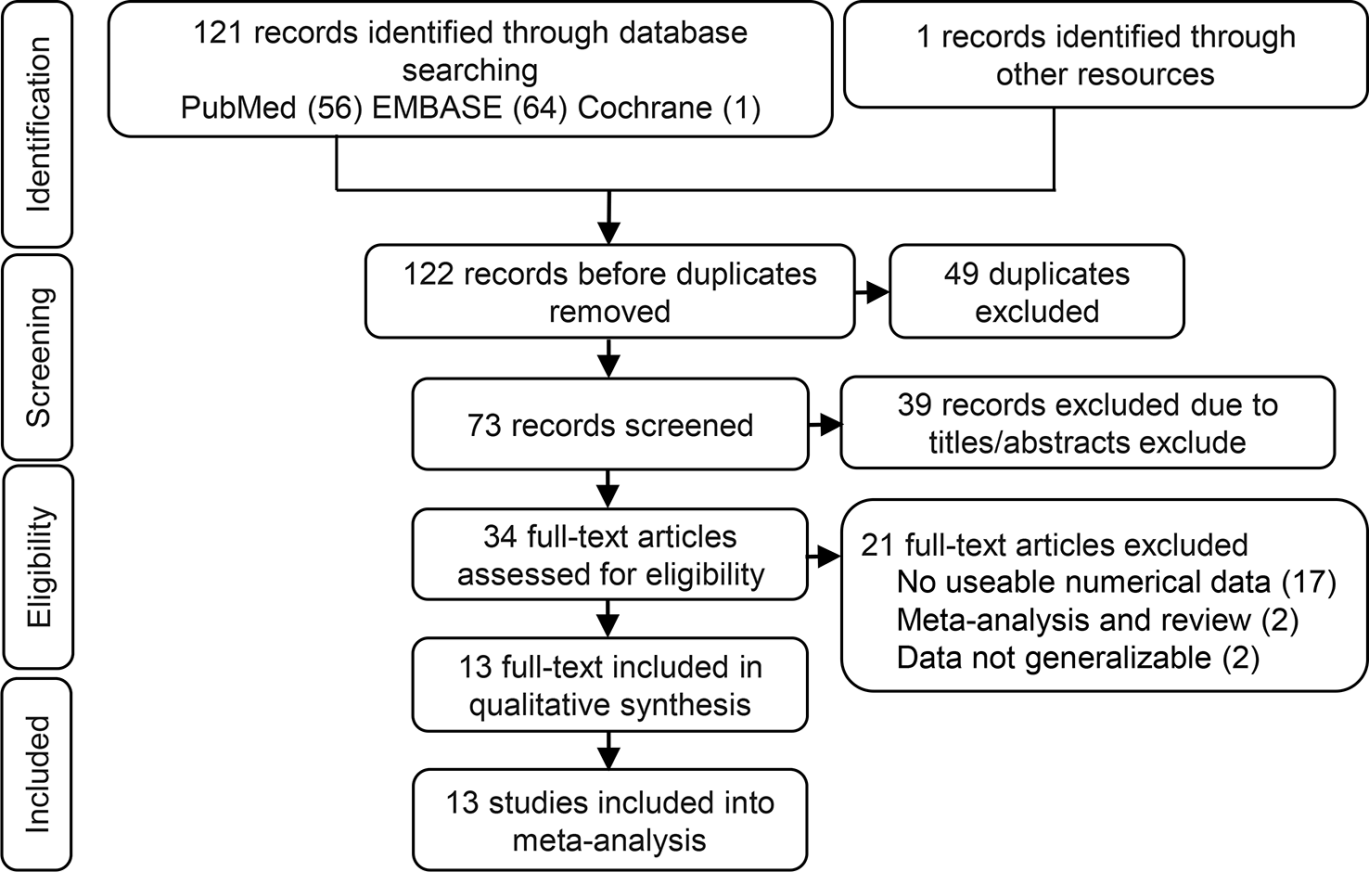
Flow chart of the identification of eligible studies.

Identification of eligible studies is summarized in **Figure 1**. We screened 122 articles for eligibility and identified 13 eligible studies. These eligible articles were published between 2014 and 2018 and included a total of 1172 participants who represented eight cancer types (**Table 1**). Most articles choose the mean and median as the cutoff value. Eight studies that used multivariate analysis of OS were included in the meta-analysis (Hou et al., 2014; Shi and Teng, 2015; Zhang et al., 2016; Zou et al., 2016; Wang et al., 2017a; Li et al., 2018a; Li et al., 2018b; Xie et al., 2018), the adjusted variables of the multivariate analysis were presented in **Table 2**. The other three studies provided survival curves (Zhang et al., 2017; Sun et al., 2018; Wei et al., 2018).

**Table 1:** Main characteristics of the 13 included studies.

Author	Year	Study design	Country	Case (N)	Type of cancer	Study period	Treatment	Disease stage	Maximum follow up (mo)	Sample type	Assay	Cut-off value	Survival end points	Analysis of OS	Adjusted variables	NOS score
Wang	2017	Retrospective single-center	China	138	Osteosarcoma	2008.01–2016.01	Received antitumor treatment	I–III	72	Tissue (-)	qRT-PCR	Median	OS, CP	Multivariate	Enneking stage, tumor size, distant metastasis, histological grade	7
Zhang	2017	Retrospective single-center	China	50	Pancreatic ductal adenocarcinoma	2006–2012	Underwent pancreaticoduodenectomy for pancreatic cancer, no chemotherapy or radiation therapy was administered before tumor excision	I–IV	62	FTT	qRT-PCR	NA	OS	Survival curve	NA	7
Han	2018	Retrospective single-center	China	105	Ovarian cancer	2013–2015	Underwent surgeries, not treated with chemotherapy or radiotherapy prior to surgery.	I–IV	NA	Tissue (-)	qRT-PCR	Median	CP	NA	NA	6
Li ZL	2018	Retrospective single-center	China	58	Cholangiocarcinoma	2010.03–2012.07	Never received chemotherapy or radiotherapy before surgical resection	I–IV	60	FTT	qRT-PCR	Median	OS, CP	Multivariate	Lymph node invasion, vascular invasion, TNM stage, postoperative recurrence	8
Hou	2014	Retrospective single-center	China	83	Lung cancer	2005–2008	NA	I–IV	99	FTT	qRT-PCR	NA	OS	Multivariate	Smoking status, TNM stage, lymphatic metastasis	7
Shi	2015	Retrospective single-center	China	84	Hepatocellular carcinoma	2006–2008	Underwent a curative hepatectomy	I–IV	60	Tissue (-)	qRT-PCR	Median	OS, CP	Multivariate	Histologic grade, TNM stage, vein invasion	7
Iranpour	2016	Retrospective single-center	Iran	38	Breast cancer	NA	NA	I–IV	NA	FTT	qRT-PCR	NA	CP	NA	NA	7
Zhang	2016	Retrospective single-center	China	132	Gastric cancer	NA	NA	I–IV	96	FTT	qRT-PCR	Median	OS, CP	Multivariate	Clinical stage, tumor depth, lymph node metastasis, distant metastasis	8
Zou	2016	Retrospective single-center	China	155	Gastric cancer	NA	Without any therapeutic before surgery	I–IV	65	Tissue (-)	qRT-PCR	Median	OS, DFS, CP	Multivariate	T stage, distant metastasis, differentiation	8
Xie	2018	Retrospective single-center	China	100	NSCLC	2010.01–2012.02	No chemotherapy or radiotherapy was received before tissue/serum collection	I–III	46	Tissue and serum	qRT-PCR	Median	OS	Multivariate	Tumor size, lymph node metastasis, TNM stage	7
Sun	2018	Retrospective single-center	China	86	Hepatocellular carcinoma	2009.11–2014.03	Underwent surgical resection	I–IV	61	FTT	qRT-PCR	mean	OS, DFS, CP	Survival curve	NA	7
Li ZH	2018	Retrospective multicenter	China	61	Pancreatic ductal adenocarcinoma	2012.01–2016.01 and 2015.07–2015.10	NA	I–IV	45	Serum	qRT-PCR	mean	OS, CP	Multivariate	Liver metastasis	8
Wei	2018	Retrospective single-center	China	82	Cholangiocarcinoma	NA	NA	I–IV	60	FTT	qRT-PCR	mean	OS, CP	Survival curve	NA	7

mo., month; NSCLC, non-small cell lung cancer; NA, not available; NOS, Newcastle-Ottawa Scale; OS, overall survival; DFS, disease-free survival; -, not mentioned; FTT, Frozen tumor tissue; q-PCR, quantitative real-time polymerase chain reaction; CP, clinical parameters; TNM, tumor, node, metastasis.

**Table 2:** The adjusted variables in the multivariate analysis of OS in the 8 included studies.

Author	Year	Clinical stage	Lymph node metastasis	Tumor differentiation	Tumor size	Vascular invasion	Tumor depth	Distant metastasis	Postoperative recurrence	Smoking status
Wang	2017	√		√	√			√		
Li ZL	2018	√	√			√			√	
Hou	2014	√	√							√
Shi	2015	√		√		√				
Zhang	2016	√	√				√	√		
Zou	2016			√			√	√		
Xie	2018	√	√		√					
Li ZH	2018							√		

OS, overall survival.

### Association Between SOX2-OT Expression and Prognosis

**Figure 2:**
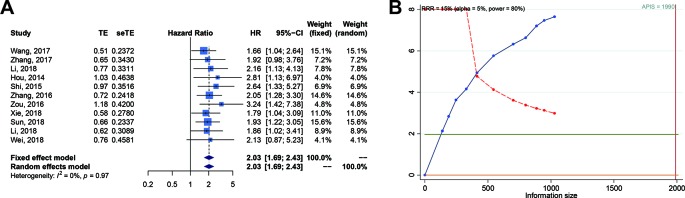
Relationship between SOX2 overlapping transcript (SOX2-OT) expression and overall survival (OS) in patients with various cancers. **(A)** Forest plot of SOX2-OT expression and OS. **(B)** trial sequential analysis (TSA) of 11 trials comparing OS of the high vs. low SOX2-OT expression. Heterogeneity adjustment required information size of 1990 participants calculated on basis of proportion of OS of 80%, RRR of 15%, α = 5%, β = 20%, power = 0.80, and I^2^ = 0%. Cumulative Z-curve crosses trial sequential monitoring boundary, showing sufficient evidence for a 15% increase in relative risk with high expression of SOX2-OT. Horizontal green lines illustrate the traditional level of statistical significance (*P* = 0.05).

We carried out a meta-analysis of the association between SOX2-OT expression and OS and DFS. The results revealed that higher SOX2-OT expression predicted an unfavorable OS (n = 11, HR = 2.026, 95% CI: [1.691–2.428], *P* < 0.0001, I^2^ = 0%) (**Figure 2A**) and a poor DFS (n = 2, HR = 2.554, 95% CI: [1.261–5.174], *P* = 0.0092, I^2^ = 66.6%, **Supplementary Figure 1**, **Table 3**). No heterogeneity was identified according to a fixed effect model (I^2^ = 0%) (**Figure 2A**). The outcomes of publication bias analysis are listed in **Table 3**.

**Table 3:** Meta-analysis of the effects of SOX2-OT overexpression on survival and clinical parameters.

Outcome	No. of trials (patients)	HR or RR(95% CI)	*P* value of Fixed-effect Model	*Z value of Fixed-effect Model*	HR or RR(95% CI)	*P* value of Random-effect Model	*Z* value of Random-effect Model	Heterogeneity I^2^(%), *P* value	*P* value of Egger’s test, Begg’s test
		Fixed-Effect estimate			Random-Effect estimate				
OS	11 (1029)	***2.026 (1.691–2.428)***	***<0.0001***	7.6500	2.026 (1.691–2.428)	<0.0001	7.6500	0.0%, 0.9698	0.0135, 0.0158
DFS	2 (241)	2.332 (1.593–3.413)	<0.0001	4.3575	***2.554 (1.261–5.174)***	***0.0092***	2.6045	66.6%, 0.0836	NA, NA
Tumor stage (**III/IV versus I/II**)	9 (784)	1.526 (1.325–1.758)	<0.0001	5.8585	***1.468 (1.106–1.949)***	***0.0079***	2.6566	71.9%, 0.0004	0.8772, 0.8348
Lymphatic metastasis (**yes versus no**)	7 (631)	1.534 (1.311–1.794)	<0.0001	5.3453	***1.554 (1.211–1.994)***	***0.0005***	3.4685	52.2%, 0.0508	0.4831, 0.8806
Distant metastasis (**yes versus no**)	4 (486)	***3.054 (1.866–4.999)***	***<0.0001***	4.4415	2.957 (1.620–5.400)	0.0004	3.5295	18.3%, 0.2989	0.1705, 0.1742
Tumor size (**large versus small**)	7 (667)	1.285 (1.118–1.478)	0.0004	3.5306	***1.264 (1.019–1.566)***	***0.0329***	2.1336	56.2%, 0.0330	0.3387, 0.2931
Depth of tumor invasion (**T3/4 versus T1/2**)	3 (369)	***1.552 (1.274–1.890)***	***<0.0001***	4.3703	1.557 (1.280–1.894)	<0.0001	4.4300	0.0%, 0.9288	0.5396, 0.6015
Differentiation (**poor/moderate versus well**)	9 (834)	1.131 (0.978–1.309)	0.0977	1.6560	1.122 (0.800–1.573)	0.5062	0.6647	78.7%, <0.0001	0.5987, 0.2971
Age (elder versus young)	10 (929)	0.981 (0.862–1.116)	0.7661	-0.2975	0.966 (0.821–1.138)	0.6812	-0.4108	31.4%, 0.1575	0.1080, 0.3970
Gender (**male versus female**)	8 (796)	1.022 (0.921–1.134)	0.6798	0.4128	1.013 (0.916–1.122)	0.7959	0.2587	0.0%, 0.8005	0.5557, 0.3223

HR, hazard ratio; RR, relative risk; CI, confidence interval; OS, overall survival; DFS, disease-free survival; NA, not available.

I^2^, index for assessing heterogeneity; value ≥25% indicates a moderate to high heterogeneity.

Egger’s test: P value of Egger’s regression for asymmetry assessment.

Begg’s test: P value of Begg and Mazumdar rank correlation test for asymmetry assessment.

Bold italics indicate statistically significant values (P < 0.05).

We performed subgroup analyses of association between SOX2-OT expression and OS using 11 studies. The results showed the presence of a significant association between SOX2-OT expression and OS when the data were fully integrated from eight studies where OS was assessed with multivariate analysis (HR = 2.052, 95% CI: [1.661; 2.536], *P* < 0.0001, I^2^ = 0%) (**Table 4**). Furthermore, a significant relationship was revealed in the subgroup analyses for OS based on sample size (*P* < 0.0001), tumor type (*P* < 0.05), sample type (*P* < 0.05), and cut-off value (*P* < 0.01).

**Table 4:** Subgroup analysis of the association between SOX2-OT overexpression and OS in patients with different cancers.

Sub variates	No. of trials	HR (95% CI) (FEM)	P value (FEM)	HR (95% CI) (REM)	P value (REM)	Heterogeneity I^2^, *P*	Heterogeneity Q	Heterogeneity tau^2^	P between subgroup (REM)
Sample size									
≥100	4	***1.942[1.486; 2.539]***	***<0.0001***	1.942[1.486; 2.539]	<0.0001	0.00%, 0.5595	2.0625	<0.0001	0.6764
≤100	7	***2.099[1.642; 2.682]***	***<0.0001***	2.099[1.642; 2.682]	<0.0001	0.00%, 0.9777	1.1828	<0.0001	
Tumor type									
Osteosarcoma	1	***1.659[1.042; 2.641]***	***0.0328***	1.659[1.042; 2.641]	0.0328	NA, 1.0000	<0.0001	NA	0.9369
Pancreatic ductal adenocarcinoma	2	***1.887[1.203; 2.959]***	***0.0057***	1.887[1.203; 2.959]	0.0057	0.00%, 0.9452	0.0047	<0.0001	
Cholangiocarcinoma	2	***2.150[1.270; 3.637]***	***0.0043***	2.150[1.270; 3.637]	0.0043	0.00%, 0.9803	0.0006	<0.0001	
Lung cancer	2	***2.019[1.265; 3.222]***	***0.0032***	2.019[1.265; 3.222]	0.0032	0.00%, 0.4068	0.6882	<0.0001	
HCC	2	***2.125[1.451; 3.113]***	***0.0001***	2.125[1.451; 3.113]	0.0001	0.00%, 0.4559	0.5558	<0.0001	
Gastric cancer	2	***2.299[1.525; 3.467]***	***0.0001***	2.299[1.525; 3.467]	0.0001	0.00%, 0.3456	0.8894	<0.0001	
Sample type									
Tissue	9	***2.080[1.699; 2.546]***	***<0.0001***	2.080[1.699; 2.546]	<0.0001	0.00%, 0.9289	3.0847	<0.0001	0.8458
Mix	1	***1.793[1.040; 3.092]***	***0.0357***	1.793[1.040; 3.092]	0.0357	NA, 1.0000	<0.0001	NA	
Serum	1	***1.860[1.015; 3.408]***	***0.0445***	1.860[1.015; 3.408]	0.0445	NA, 1.0000	<0.0001	NA	
Cut-off value									
Median	6	***2.040[1.616; 2.575]***	***<0.0001***	2.040[1.616; 2.575]	<0.0001	0.00%, 0.7362	2.7648	<0.0001	0.9231
others	2	***2.196[1.279; 3.771]***	***0.0043***	2.196[1.279; 3.771]	0.0043	0.00%, 0.5099	0.4343	<0.0001	
mean	3	***1.935[1.379; 2.714]***	***0.0001***	1.935[1.379; 2.714]	0.0001	0.00%, 0.9702	0.0604	<0.0001	
Analysis model									
Multivariate	8	***2.052[1.661; 2.536]***	***<0.0001***	2.052[1.661; 2.536]	<0.0001	0.00%, 0.8533	3.3257	<0.0001	0.8178
Survival curve	3	***1.956[1.380; 2.773]***	***0.0002***	1.956[1.380; 2.773]	0.0002	0.00%, 0.9798	0.0408	<0.0001	

HR, hazard ratio; CI, confidence interval; OS, overall survival; HCC, hepatocellular carcinoma; FEM, fixed-effect model; REM, random-effect model; NA, not available.

I^2^, index for assessing heterogeneity; value ≥25% indicates a moderate to high heterogeneity.

Bold italics indicate statistically significant values (P < 0.05).

Eight studies employed Cox multivariate analysis to survey the prognostic value of lncRNA SOX2-OT expression on the prognosis of cancer patients (Hou et al., 2014; Shi and Teng, 2015; Zhang et al., 2016; Zou et al., 2016; Wang et al., 2017a; Li et al., 2018a; Li et al., 2018b; Xie et al., 2018). An in-depth subgroup analysis is required to clearly define the values of the adjusted variables in multivariate analysis (**Table 5**). Subgroup analysis stratified by independent prognostic factors, such as clinical stage (*P* < 0.0001), lymph node metastasis (*P* < 0.0001), tumor differentiation (*P* < 0.0001), tumor size (*P* < 0.01), vascular invasion (*P* < 0.001), tumor depth (*P* < 0.001), distant metastasis (*P* < 0.0001), postoperative recurrence (*P* < 0.05), and smoking status (*P* < 0.05) (**Table 5**) demonstrated that a significant relationship existed between lncRNA SOX2-OT expression and OS.

**Table 5:** Subgroup analyses of the OS in the eight included studies based on adjusted variables.

Sub variates	No. of trials	HR (95% CI)	P value (FEM)	HR (95% CI)	P value (REM)	Heterogeneity I^2^, *P*	Heterogeneity Q	Heterogeneity tau^2^	P between subgroup
		(FEM)		(REM)					(REM)

Clinical stage									
YES	6	***2.007[1.587; 2.538]***	***<0.0001***	2.007[1.587; 2.538]	<0.0001	0.00%, 0.8483	2.0058	<0.0001	0.8855
NO	2	***2.260[1.388; 3.681]***	***0.0010***	2.283[1.350; 3.859]	0.0021	11.85%, 0.2868	1.1344	0.0183	
Lymph node metastasis									
YES	4	***2.060[1.532; 2.771]***	***<0.0001***	2.060[1.532; 2.771]	<0.0001	0.00%, 0.8694	0.7161	<0.0001	0.9731
NO	4	***2.044[1.511; 2.765]***	***<0.0001***	2.044[1.511; 2.765]	<0.0001	0.00%, 0.4561	2.6082	<0.0001	
Tumor differentiation									
YES	3	***2.109[1.488; 2.990]***	***<0.0001***	2.174[1.454; 3.251]	0.0002	19.49%, 0.2888	2.4842	0.0263	0.9251
NO	5	***2.020[1.548; 2.636]***	***<0.0001***	2.020[1.548; 2.636]	<0.0001	0.00%, 0.9378	0.8047	<0.0001	
Tumor size									
YES	2	***1.714[1.204; 2.441]***	***0.0028***	1.714[1.204; 2.441]	0.0028	0.00%, 0.8317	0.0452	<0.0001	0.4485
NO	6	***2.269[1.742; 2.955]***	***<0.0001***	2.269[1.742; 2.955]	<0.0001	0.00%, 0.8851	1.7300	<0.0001	
Vascular invasion									
YES	2	***2.375[1.481; 3.810]***	***0.0003***	2.375[1.481; 3.810]	0.0003	0.00%, 0.6755	0.1753	<0.0001	0.7737
NO	6	***1.978[1.562; 2.507]***	***<0.0001***	1.978[1.562; 2.507]	<0.0001	0.00%, 0.7476	2.6905	<0.0001	
Tumor depth									
YES	2	***2.299[1.525; 3.467]***	***0.0001***	2.299[1.525; 3.467]	0.0001	0.00%, 0.3456	0.8894	<0.0001	0.7971
NO	6	***1.970[1.539; 2.521]***	***<0.0001***	1.970[1.539; 2.521]	<0.0001	0.00%, 0.8442	2.0359	<0.0001	
Distant metastasis									
YES	4	***1.965[1.493; 2.585]***	***<0.0001***	1.965[1.493; 2.585]	<0.0001	0.00%, 0.5739	1.9927	<0.0001	0.8639
NO	4	***2.188[1.569; 3.050]***	***<0.0001***	2.188[1.569; 3.050]	<0.0001	0.00%, 0.7786	1.0935	<0.0001	
Postoperative recurrence									
YES	1	***2.160[1.129; 4.133]***	***0.0200***	2.160[1.129; 4.133]	0.0200	NA, 1.0000	<0.0001	NA	0.9609
NO	7	***2.040[1.631; 2.551]***	***<0.0001***	2.040[1.631; 2.551]	<0.0001	0.00%, 0.7705	3.2990	<0.0001	
Smoking status									
YES	1	***2.808[1.131; 6.969]***	***0.0260***	2.808[1.131; 6.969]	0.0260	NA, 1.0000	<0.0001	NA	0.7648
NO	7	***2.016[1.622; 2.506]***	***<0.0001***	2.016[1.622; 2.506]	<0.0001	0, 0.8283	2.8426	<0.0001	

HR, hazard ratio; CI, confidence interval; OS, overall survival; HCC, hepatocellular carcinoma; FEM, fixed-effect model; REM, random-effect model; NA, not available; YES, this clinicopathology parameters is the adjusted variable for OS in the included studies; NO: this clinicopathology parameters is not the adjusted variable for OS in the included studies.

I^2^, index for assessing heterogeneity; value ≥25% indicates a moderate to high heterogeneity.

Bold italics indicate statistically significant values (P <0.05).

### Correlation Between SOX2-OT Expression and Clinicopathological Characteristics

**Figure 3:**
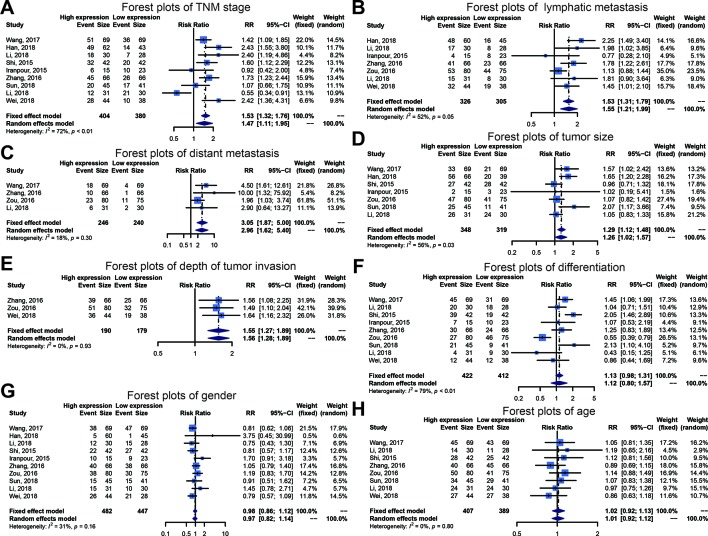
Forest plots of main clinical parameters under the upregulation or downregulation of SOX2 overlapping transcript (SOX2-OT). **(A)** tumor, node, metastasis (TNM) stage, **(B)** lymphatic metastasis, **(C)** distant metastasis, **(D)** tumor size, **(E)** depth of tumor invasion, **(F)** differentiation, **(G)** gender, and **(H)** age.

We executed an analysis of the association between SOX2-OT expression and clinicopathological characteristics (**Table 3**). The results indicated that overexpression of SOX2-OT was significantly correlated with TNM stage. Higher SOX2-OT expression was associated with high TNM stage for several malignancies (n = 9, RR = 1.468; 95% CI: [1.106–1.949], *P* = 0.0079, I^2^ = 71.9%, **Figure 3A**). SOX2-OT expression was significantly correlated with lymphatic metastasis (n = 7, RR = 1.554, 95% CI: [1.211–1.994], *P* = 0.0005, I^2^ = 52.2%, **Figure 3B**), distant metastasis (n = 4, RR = 3.054, 95% CI: [1.866–4.999], *P* < 0.0001, I^2^ = 18.3%, **Figure 3C**), tumor size (n = 7, RR = 1.264, 95% CI: [1.019–1.566], *P* < 0.0329, I^2^ = 56.2%, **Figure 3D**), depth of tumor invasion (n = 3, RR = 1.552, 95% CI: [1.274–1.890], *P* < 0.0001, I^2^ = 0.0%, **Figure 3E**). However, SOX2-OT expression was not correlated with differentiation (n = 9, RR = 1.122, 95% CI: [0.800–1.573], *P* = 0.5062, I^2^ = 78.7%, **Figure 3F**), gender (n = 8, RR = 1.022, 95% CI: [0.921–1.134], *P* = 0.6798, I^2^ = 0.0%, **Figure 3G**), or age (n = 10, RR = 0.966, 95% CI: [0.821–1.138], *P* = 0.6812, I^2^ = 31.4%, **Figure 3H**).

In order to examine the robustness of OS, the trial sequencing monitoring boundaries executed to the meta-analysis supposed a decrease in relative risk by 15%. The cumulative Z-curve crossed the trial sequential monitoring boundary for benefit, indicating that sufficient evidence exists for a 15% relative risk reduction (RRR) when SOX2-OT expression is low (**Figure 2B**).

**Figure 4:**
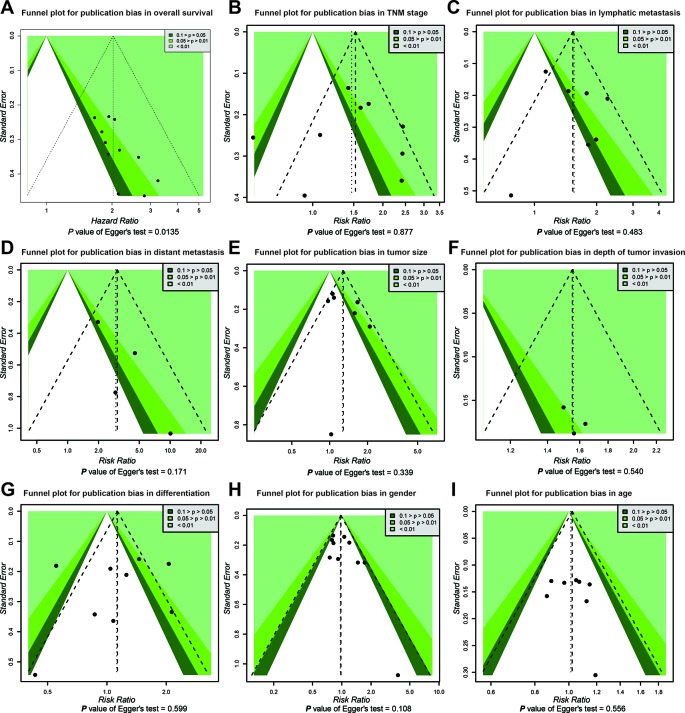
Funnel plot for publication bias in overall survival and clinicopathological characteristics. **(A)** overall survival (OS), **(B)** tumor, node, metastasis (TNM) stage, **(C)** lymphatic metastasis, **(D)** distant metastasis, **(E)** tumor size, **(F)** depth of tumor invasion, **(G)** differentiation, **(H)** gender, and **(I)** age.

Publication bias of the association between SOX2-OT expression and prognosis was inferred based on our Egger’s test (*P* < 0.05) (**Figure 4A**). No distinct biases of the correlation between SOX2-OT expression and clinicopathological characteristics were found across included studies on the basis of funnel plots and the *P* value of the Egger’s test (**Figures 4B–I**).

### Meta-Regression and Stratified Analysis

**Figure 5:**
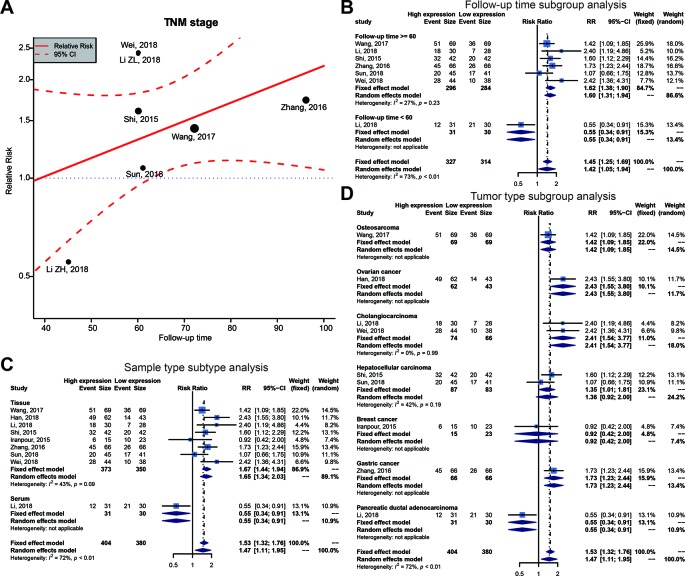
Meta-regression plot and subgroup analysis of tumor, node, metastasis (TNM) stage and follow-up time, sample type and tumor type. **(A)** Meta-regression plot correction of follow-up time and TNM stage. From the meta-regression plot correction, we determined that a follow-up time of more than 60 months correlated with higher TNM stage. The point of determination for differences in TNM stage is a follow-up time of about 60 months. **(B)** Follow-up time subgroup, **(C)** sample type subtype, and **(D)** tumor type subtype.

To investigate the possible sources of heterogeneity, we gathered the original articles for subgroup analyses, based on various factors. **Table 6** displays the outcomes of a meta-regression that examined the source of high heterogeneity for TNM stage. The follow-up time, sample type, and tumor type could significantly explain heterogeneity for survival outcomes in the *post-hoc* analysis (**Table 6**, **Figure 5A**). On the basis of the results of the meta-regression, we carried out a subgroup analysis on groups of patients with the follow-up time, sample type, and tumor type (**Figures 5B–D**). This subgroup analysis showed a significantly lower heterogeneity in the above 60 months follow-up group, the tissue group, or the Cholangiocarcinoma group, which suggested that the relationship between high SOX2-OT expression and TNM stage has stronger efficacy in these groups.

**Table 6:** Meta-regression analysis of heterogeneity in TNM staging.

Moderators	Variables of regression	HR_interaction_ (95% CI)	*P* value of regression	I^2^	Cochrane Q (*P* value)
Year	Year	1.025(0.799–1.315)	0.8453	75.16%	0.0002
Sample size	Sample size	1.005(0.996–1.015)	0.3015	72.91%	0.0005
Follow up	Follow up	***3.399(1.915–6.035)***	***<0.0001***	0.00%	0.3743
Country	Intercept	1.524(1.134–2.049)	0.0052	73.86%	0.0004
	Iran	0.604(0.204–1.788)	0.3623	73.86%	0.0004
Sample size	Intercept	1.780(1.116–2.840)	0.0155	72.38%	0.0007
	Less than 100	0.728(0.400–1.325)	0.2993	72.38%	0.0007
Tumor type	Intercept	0.920(0.424–1.998)	0.8331	0.00%	0.4329
	Cholangiocarcinoma	***2.621(1.071–6.412)***	***0.0348***	0.00%	0.4329
	Gastric cancer	1.881(0.806–4.390)	0.1438	0.00%	0.4329
	Hepatocellular carcinoma	1.511(0.660–3.458)	0.3283	0.00%	0.4329
	Osteosarcoma	1.540(0.678–3.495)	0.3020	0.00%	0.4329
	Ovarian cancer	***2.638(1.077–6.464)***	***0.0338***	0.00%	0.4329
	Pancreatic ductal adenocarcinoma	0.601(0.239–1.513)	0.2799	0.00%	0.4329
Sample type	Intercept	0.553(0.297–1.029)	0.0614	42.03%	0.0981
	Tissue	***2.976(1.547***–***5.725)***	***0.0011***	42.03%	0.0981
cut off value	Intercept	1.094(0.685–1.747)	0.7071	69.34%	0.0033
	Median	1.646(0.926–2.927)	0.0895	69.34%	0.0033

HR_interaction_, interaction effect calculated by meta-regression; Positive direction indicates that possible moderators might strengthen OS in the SOX2-OT overexpression relative to underexpression.

Bold italics indicate statistically significant values (P < 0.05).

Meta-regression analysis (**Supplementary Table 3**) and stratified analysis (**Supplementary Table 4**) did not demonstrate heterogeneity between all potential factors and the other clinical parameters.

### Validation by Independent TCGA Datasets

To validate the results of the meta-analysis, we employed tissue SOX2-OT expression data and the matching survival data from TCGA datasets. The results indicated that high SOX2-OT expression in tissues was not associated with worse OS in the pooled analysis of TCGA datasets for all the tumors (n = 32, HR = 1.078, 95% CI 0.922–1.262, *P* = 0.346, I^2^ = 66.3%) (**Table 7**, **Supplementary Figure 2**), which included 9676 patients with diversified types of cancer.

**Table 7:** HRs and corresponding 95% CIs of SOX2-OT overexpression in tumors based on The Cancer Genome Atlas (TCGA) datasets.

	OS	
	HR (95% CI)	*P* Value
TCGA-LAML	1.062(0.681–1.656)	0.789
TCGA-ACC	**0.407(0.192–0.862)**	**0.017**
TCGA-BLCA	1.317(0.98–1.769)	0.064
TCGA-BRCA	**1.481(1.033–2.123)**	**0.02**
TCGA-CESC	**0.557(0.351–0.885)**	**0.014**
TCGA-CHOL	0.918(0.364–2.319)	0.856
TCGA-COAD	1.403(0.94–2.093)	0.109
TCGA-ESCA	0.744(0.453–1.22)	0.248
TCGA-HNSC	0.995(0.762–1.298)	0.97
TCGA-KICH	0.86(0.233–3.181)	0.822
TCGA-KIRC	**1.567(1.157–2.121)**	**0.003**
TCGA-GBM	NA	NA
TCGA-KIRP	0.815(0.451–1.473)	0.5
TCGA-LIHC	1.467(0.845–2.548)	0.24
TCGA-LUAD	**0.738(0.552–0.988)**	**0.04**
TCGA-LUSC	0.79(0.603–1.035)	0.085
TCGA-DLBC	4.429(0.509–38.56)	0.059
TCGA-MESO	**0.567(0.352–0.913)**	**0.013**
TCGA-OV	0.921(0.711–1.193)	0.53
TCGA-PAAD	0.89(0.591–1.339)	0.574
TCGA-PCPG	2.648(0.526–13.329)	0.231
TCGA-PRAD	0.541(0.155–1.883)	0.362
TCGA-READ	1.541(0.711–3.337)	0.29
TCGA-SARC	**1.664(1.03–2.69)**	**0.042**
TCGA-SKCM	0.642(0.31–1.329)	0.233
TCGA-STAD	**1.82(1.195–2.771)**	**0.022**
TCGA-TGCT	2.269(0.314–16.419)	0.455
TCGA-THYM	**7.349(1.494–36.153)**	**0.001**
TCGA-THCA	**3.954(0.929–16.837)**	**0.004**
TCGA-UCS	**2.393(1.012–5.656)**	**0.03**
TCGA-UCEC	**2.142(1.145–4.004)**	**0.002**
TCGA-UVM	1.461(0.645–3.311)	0.365
TCGA-LGG	**0.662(0.465–0.941)**	**0.019**

The data were subjected to the Kaplan-Meier method and log-rank test.

HR, hazard ratio; CI, confidence interval; OS, overall survival; NA, not available.

Bold indicate statistically significant values (P < 0.05).

ACC, adrenocortical cancer; BLCA, bladder cancer; BRCA, breast cancer; CESC, cervical cancer; CHOL, bile duct cancer; COAD, colon cancer; DLBC, diffuse Large B-cell Lymphoma; ESCA, esophageal cancer; GBM, glioblastoma multiforme; HNSC, head and neck squamous cell carcinoma; KICH, kidney Chromophobe; KIRC, kidney renal clear cell carcinoma; KIRP, kidney renal papillary cell carcinoma; LAML, acute myeloid leukemia; LGG, glioma, LIHC, liver cancer; LUAD, lung adenocarcinoma; LUSC, lung squamous cell carcinoma; MESO, mesothelioma; OV, ovarian cancer; PAAD, pancreatic cancer; PCPG, pheochromocytoma and paraganglioma; PRAD, prostate cancer; READ, rectum adenocarcinoma; SARC, sarcoma; SKCM, melanoma; STAD, gastric cancer; TGCT, testicular tumors; THCA, thyroid cancer; THYM, thymoma; UCEC, endometrioid cancer; UCS, uterine carcinosarcoma; UVM, uveal melanoma.

**Figure 6:**
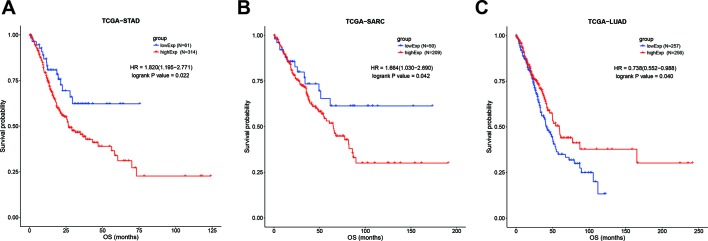
Kaplan-Meier survival curves for the overall survival of cancer patients, stratified by SOX2-OT expression levels. **(A)** TCGA-STAD, **(B)** TCGA−SARC, **(C)** TCGA−LUAD. STAD, gastric cancer; SARC, sarcoma; LUAD, lung adenocarcinoma.

However, focusing on single tumor types combined with meta-analysis revealed that upregulation of SOX2-OT was significantly associated with worse OS in sarcoma (TCGA-SARC; HR = 1.664, 95% CI 1.03–2.69; *P* = 0.042, **Figure 6A**) and gastric cancer (TCGA-STAD; HR = 1.82, 95% CI 1.195–2.771; *P* = 0.022, **Figure 6B**), while the association was opposite in lung adenocarcinoma (TCGA-LUAD; HR = 0.738, 95% CI 0.552–0.988; *P* = 0.04) (**Figure 6C**). In the other tumor types, SOX2-OT expression was not associated with worse OS (**Table 7**, **Supplementary Figures 3** **and** **4**).

### Functional Analysis of SOX2-OT Related Genes in Human Tumors

**Figure 7:**
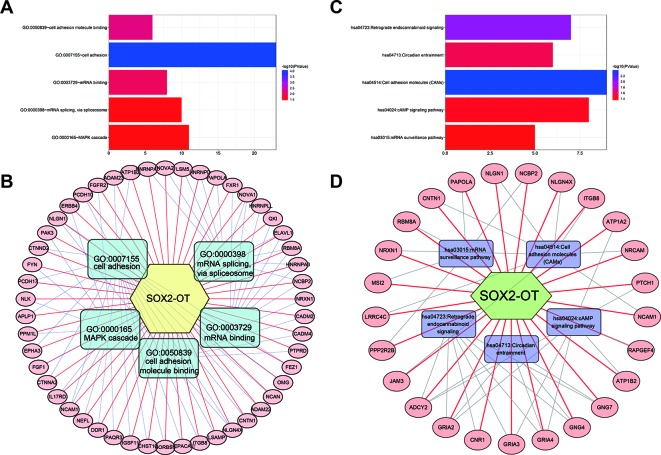
Significantly enriched gene ontology (GO) categories and Kyoto Encyclopedia of Genes and Genomes (KEGG) pathways of potential targets of long non-coding RNAs (lncRNA) SOX2 overlapping transcript (SOX2-OT) in cancer patients. **(A)** biological processes (BP), **(B)** the lncRNA SOX2-OT-GO-mRNA network was generated based on the Multi Experiment Matrix (MEM) and DAVID databases. **(C)** KEGG pathway. **(D)** the lncRNA SOX2-OT-KEGG-mRNA network was generated based on the MEM and DAVID databases.

To systematically analyze the underlying gene regulatory mechanisms of SOX2-OT, a total of 500 target genes were identified with Multi Experiment Matrix (MEM) (**Supplementary Figure 5**). GO and KEGG analyses were executed. Validated target genes of SOX2-OT enriched GO terms including cell adhesion, cell adhesion molecule (CAM) binding, mRNA binding, mRNA splicing *via* spliceosome, and MAPK cascade (**Figure 7A**). These relevant GO terms were considered as the most specific and useful for describing the concrete function of SOX2-OT. The visualization network is shown in **Figure 7B**. Furthermore, KEGG enrichment analysis indicated that SOX2-OT may play a critical role in cancers *via* several pathways including CAMs, retrograde endocannabinoid signaling, circadian entrainment, cAMP signaling pathway, and mRNA surveillance pathway (**Figure 7C**). These corresponding KEGG terms were considered as the most specific and useful for describing the concrete pathway of SOX2-OT. The visualization network is presented in **Figure 7D**.

## Discussion

Several studies have indicated that high expression of SOX2-OT is significantly related with the prognosis and clinicopathological outcomes in cancers (Hou et al., 2014; Shi and Teng, 2015; Iranpour et al., 2016; Zhang et al., 2016; Zou et al., 2016; Wang et al., 2017a; Wang et al., 2017b; Han et al., 2018; Li et al., 2018a; Li et al., 2018b; Sun et al., 2018; Wei et al., 2018; Xie et al., 2018). The crucial role that SOX2-OT may play in the progression of many cancers had been further outlined in reviews (Shahryari et al., 2015; Castro-Oropeza et al., 2018). A meta-analysis by Jing et al. proposed that the overexpression of SOX2-OT indicated higher TNM stage and a worse OS in cancer patients, but failed to predict distant metastasis and lymph node metastasis in Chinese cancer patients (Jing et al., 2017). Moreover, other studies since 2014 have investigated the relationship between SOX2-OT and the prognosis of cancer patients (Hou et al., 2014; Shi and Teng, 2015; Iranpour et al., 2016; Zhang et al., 2016; Zou et al., 2016; Wang et al., 2017a; Wang et al., 2017b; Han et al., 2018; Li et al., 2018a; Li et al., 2018b; Sun et al., 2018; Wei et al., 2018; Xie et al., 2018). The present study was performed to obtain a more definite conclusion and assess the potential mechanisms of SOX2-OT effects by integrating the outcomes of published studies and TCGA survival data and running GO and KEGG analyses.

The present meta-analysis of a combination of 1172 patients from 13 eligible studies with 9676 patients from TCGA investigated thoroughly the correlations between elevated expression of SOX2-OT and prognosis as well as clinicopathological outcomes in cancer patients. The NOS was applied to evaluate the quality of all the selected studies, and Egger’s test and Begg’s test were used to examine the publication bias. If the *P* value of the Egger’s test was less than 0.05, we also checked the reliability of the results by TSA.

Our results indicated that elevated expression of SOX2-OT was significantly related to worse prognosis indicators, with an OS of 2.026 (95% CI: 1.691–2.428), and a DFS of 2.554 (95% CI: 1.261–5.174). Regarding the clinicopathological characteristics of patients with cancers, our research suggested that high SOX2-OT expression was significantly associated with the invasion of cancers, as reveal by the tumor stage (RR = 1.468, 95% CI: 1.106–1.949), lymphatic metastasis (RR = 1.554, 95% CI: 1.211–1.994), distant metastasis (RR = 3.054, 95% CI: 1.866–4.999), tumor size (RR = 1.264, 95% CI: 1.019–1.566), and depth of tumor invasion (RR = 1.552, 95% CI: 1.274–1.890), but couldn’t predict histological differentiation, age, or gender.

According to our findings, SOX2-OT shows the potential to be used as a marker for progression and prognosis. A subgroup analysis indicated that elevated SOX2-OT expression was substantially associated with OS in sarcoma (SARC) and gastric cancer (STAD) patients, according to the publications and the TCGA datasets. As for pancreatic cancer (PAAD), bile duct cancer (CHOL), lung adenocarcinoma (LUAD), and lung squamous cell carcinoma (LUSC), SOX2-OT overexpression was correlated with a bad prognosis in the publications. However, in the TCGA datasets, SOX2-OT was associated with a good prognosis although the results were not statistically significant; the corresponding HR values were 0.89 (95% CI: 0.591–1.339, *P* = 0.574), 0.918 (95% CI: 0.364–2.319, *P* = 0.856), 0.738 (95% CI: 0.552–0.988, *P* = 0.04), and 0.79 (95% CI: 0.603–1.035, *P* = 0.085), respectively. High expression of SOX2-OT in liver cancer (LIHC) in the TCGA datasets was correlated with an unfavorable prognosis (HR = 1.467, 95% CI: 0.845–2.548, *P* = 0.24) although the results were not statistically significant, which was consistent with the publications (Shi and Teng, 2015; Sun et al., 2018) (**Tables 4** and **7**). Kaplan-Meier analysis initially suggested that SOX2-OT overexpression was associated with a bad OS in adrenocortical cancer (ACC), cervical cancer (CESC), mesothelioma (MESO), and glioma (LGG), and associated with a worse OS in breast cancer (BRCA), kidney renal clear cell carcinoma (KIRC), thymoma (THYM), thyroid cancer (THCA), uterine carcinosarcoma (UCS), and endometrioid cancer (UCEC) according to the TCGA datasets (**Table 7** and **Supplementary Figures 3** and **4**). Sampling error and publication bias may explain the inconsistent results between literature studies and studies on TCGA datasets.

Heterogeneity appeared in the clinicopathological aspects including tumor stage, lymphatic metastasis, and tumor size (*P* < 0.1). Since the presence of heterogeneity may affect the results of the meta-analysis, the heterogeneity has been dealt cautiously with a random effects model in order to reduce the effect of heterogeneity on the merged results. Publication bias was prominent in studies with OS data (*P* < 0.05) as showed by the Egger’s, Begg’s test, and funnel plots. Hence, the TSA data suggested the results of our study were statistically stable.

Recently, studies on the functioning of SOX2-OT in cancer have spread and cumulative evidence indicating that SOX2-OT could affect various biological behaviors of numerous tumors. Li et al. pointed out that SOX2-OT competitively binds to the miR-200 family to regulate the expression of SOX2, and SOX2-OT promotes epithelial-mesenchymal transition (EMT) and stem cell-like properties by regulating SOX2 expression, thereby promoting invasion and metastasis of pancreatic duct adenocarcinoma (Li et al., 2018a). Qu et al. proposed that SOX2-OT was highly expressed in gastric cancer cells, which promoted the expression of AKT2 by targeting miR-194-5p, thus elevating cell proliferation and metastasis (Qu and Cao, 2018). Finally, Wei et al. discovered that the upregulation of lncRNA SOX2-OT by transcription factor IRF4 promotes cell proliferation and metastasis in cholangiocarcinoma *via* upregulating SOX2, and activates PI3K/AKT signaling pathway *via* suppressing the nuclear transcription of PTEN (Wei et al., 2018).

The exact gene regulatory mechanisms of SOX2-OT remain poorly understood. Therefore, we uncovered the validated targeting genes of SOX2-OT through the MEM platform, and a comprehensive target gene network analysis was performed. The GO and KEGG pathway analysis together revealed that some CAMs and pathways may be regulated by SOX2-OT. SOX2-OT appears to play a critical role in the cancers *via* different pathways, including mRNA binding and mRNA splicing, similar to the post-transcriptional regulating functions of other lncRNAs. The above findings suggest that the elevation of SOX2-OT expression is associated with the processes of tumor invasion and metastasis, consistent with our findings.

Our study is consistent with the most recent study by Song et al. in which lncRNA SOX2-OT overexpression was significantly correlated with worse OS and more advanced clinical stages of solid tumors based on 943 cases from 10 studies, all of them being Asians (Song et al., 2018). Consistently, analysis of 481 patients from five studies by Jing et al. showed that high SOX2-OT expression predicted poor OS and more advanced tumor progression, but failed to predict distant metastasis and lymph node metastasis in Chinese cancer patients (Jing et al., 2017). Herein, we have performed a more comprehensive study on the clinicopathological significance of SOX2-OT expression in cancer patients. First, we included 13 eligible articles involving 1172 cancer patients and 32 TCGA cancer datasets involving 9676 cancer patients to investigate a total of 10,848 participants in our study. Second, we investigated both clinicopathological and prognostic significance of SOX2-OT expression based on comprehensive clinical data and performed a series of subgroup analyses based on prognostic types, adjusted variables in the multivariate analysis of OS, sample sizes, cancer types, sample types, cut-off values, analysis models, and clinicopathological characteristics. These stratifications increase our understanding of the clinicopathological significance of SOX2-OT expression in cancers. Third, TSA on the applicable literature was used to investigate reliability and conclusiveness of available evidence for the prognostic significance of SOX2-OT expression. Fourth, the prognostic value was validated using TCGA datasets and the potential functions were explored using GO and KEGG.

In this particular study, there were some limitations. As to this meta-analysis, different cut-off values and sample types of the selected articles contributed publication bias. Since direct results of survival analysis were unavailable, a divergence in HR values might significantly contribute to extract the survival data through the Kaplan-Meier curve. Consequently, in-depth study is required to investigate the clinical value and prognosis significance of SOX2-OT in cancers.

In order to increase the sample size, we used TCGA datasets for further analysis and validation, but only the results of gastric cancer and sarcoma were consistent with those based the publications. In order to clarify the mechanism by which SOX2-OT is involved in gastric cancer and sarcoma, further molecular biology experiment is warranted to explore other possible signaling pathways or target molecules.

In conclusion, our report shows that elevated SOX2-OT expression was significantly related with invasion and metastasis progress in cancers, implying shorter OS and DFS, a poorer TNM stage, higher rates of lymphatic and distant metastasis, larger tumor size, and deeper invasion. We also concluded that SOX2-OT plays a crucial role *via* a few pathways. Considering the limitations, further studies are necessary in order to better define the functions of SOX2-OT in cancers.

## Author Contributions

Participated in research design: YL, MD, MS, and DH. Performed data analysis: MS, SW, MD, JZ, PL, XW, DW, JZ, DC, and JL. Wrote or contributed to the writing of the manuscript: MS and HL.

## Funding

This research was supported by the National Natural Science Foundation of China (81902498), Natural Science Foundation of Hubei Province of China (2019CFB177), Natural Science Foundation of Hubei Provincial Department of Education (Q20182105), Chen Xiao-ping Foundation for the development of science and technology of Hubei Provincial (CXPJJH11800001-2018333), Natural Science Foundation of Hubei Province of China (2016CFB530) and Faculty Development Foundation of Hubei University of Medicine (2014QDJZR01), and National Students’ platform for innovation and entrepreneurship training program (201810929005, 201810929009, 201810929068, and 201813249010).

## Conflict of Interest

The authors declare that the research was conducted in the absence of any commercial or financial relationships that could be construed as a potential conflict of interest.

## Abbreviations

SOX2-OT, SOX2 overlapping transcript; lncRNA, Long non-coding RNAs; RNA-seq, RNA deep sequencing; TCGA, The Cancer Genome Atlas; OS, Overall survival; GO, Gene ontology; KEGG, Kyoto Encyclopedia of Genes and Genomes; MEM, Multi Experiment Matrix; HR, Hazard ratio; RR, Risk ratio; CI, Confidence interval; TSA, Trial sequential analysis; DFS, Disease free survival; TNM, Tumor, node, metastasis; q-RTPCR, Quantitative real-time polymerase chain reaction; NOS, Newcastle-Ottawa Scale; MOOSE, Meta-analysis of Observational Studies in Epidemiology; NA, Not available; ACC, Adrenocortical cancer; BLCA, Bladder cancer; BRCA, Breast cancer; CESC, Cervical cancer; CHOL, Bile duct cancer; COAD, Colon cancer; DLBC, Diffuse large B-cell lymphoma; ESCA, Esophageal cancer; GBM, Glioblastoma multiforme; HNSC, Head and neck squamous cell carcinoma; KICH, Kidney chromophobe; KIRC, Kidney renal clear cell carcinoma; KIRP, Kidney renal papillary cell carcinoma; LAML, Acute myeloid leukemia; LGG, Glioma; LIHC, Liver cancer; LUAD, Lung adenocarcinoma; LUSC, Lung squamous cell carcinoma; MESO, Mesothelioma; OV, Ovarian cancer; PAAD, Pancreatic cancer; PCPG, Pheochromocytoma and paraganglioma; PRAD, Prostate cancer; READ, Rectum adenocarcinoma; SARC, Sarcoma; SKCM, Melanoma; STAD, Gastric cancer; TGCT, Testicular tumors; THCA, Thyroid cancer; THYM, Thymoma; UCEC, Endometrioid cancer; UCS, Uterine carcinosarcoma; UVM, Uveal melanoma; CAMs, Cell adhesion molecules; EMT, Epithelial-mesenchymal transition; CP, Clinical parameters; BP, Biological processes.

## Supplementary Material

Supplementary Figure 1 Relationship of SOX2-OT expression and DFS in various cancers.

Supplementary Figure 2 Meta-analysis of TCGA datasets estimating the association of SOX2-OT with the patients’ overall survival (OS).

Supplementary Figure 3 Kaplan-Meier survival curves for overall survival of 16 cancers in TCGA, stratified by SOX2-OT expression levels. **(A)** ACC, **(B)** BLCA, **(C)** BRCA, **(D)** CESC, **(E)** CHOL, **(F)** COAD, **(G)** DLBC, **(H)** ESCA, **(I)** HNSC, **(J)** KICH, **(K)** KIRC, **(L)** KIRP, **(M)** LAML, **(N)** LGG, **(O)** LIHC, **(P)** LUAD.

Supplementary Figure 4 Kaplan-Meier survival curves for overall survival of the other 16 cancers in TCGA, stratified by SOX2-OT expression levels. **(A)** LUSC, **(B)** MESO, **(C)** OV, **(D)** PAAD, **(E)** PCPG, **(F)** PRAD, **(G)** READ, **(H)** SARC, **(I)** SKCM, **(J)** STAD, **(K)** TGCT, **(L)** THCA, **(M)** THYM, **(N)** UCEC, **(O)** UCS, **(P)** UVM.

Supplementary Figure 5 Totally 500 target genes of SOX2-OT were identified with Multi Experiment Matrix (MEM).

Supplementary Table 1 Databases searching terms. **(A)** Search criterion of Medline (via PubMed, from inception to Jan 1st, 2019) (n=47), **(B)** Search criterion of Embase (from 1966 to Jan 20, 2019) (n=49), **(C)** Search criterion of Cochrane Library (Jan 1st, 2019) (n=1).

Supplementary Table 2 Results of quality assessment using the Newcastle–Ottawa Scale for the included studies.

Supplementary Table 3 Meta-regression analysis of heterogeneity for clinicopathological parameters in cancers.

Supplementary Table 4 Subgroup analysis for SOX2-OT overexpression and clinicopathological parameters in cancers.
