# Supplementary figures and images for: Clinicopathological Implication of Long Non-Coding RNAs SOX2 Overlapping Transcript and Its Potential Target Gene Network in Various Cancers
Source: Front Genet. 2020 Jan 23;10:1375. doi: 10.3389/fgene.2019.01375 (PMC6989546; doi:10.3389/fgene.2019.01375)

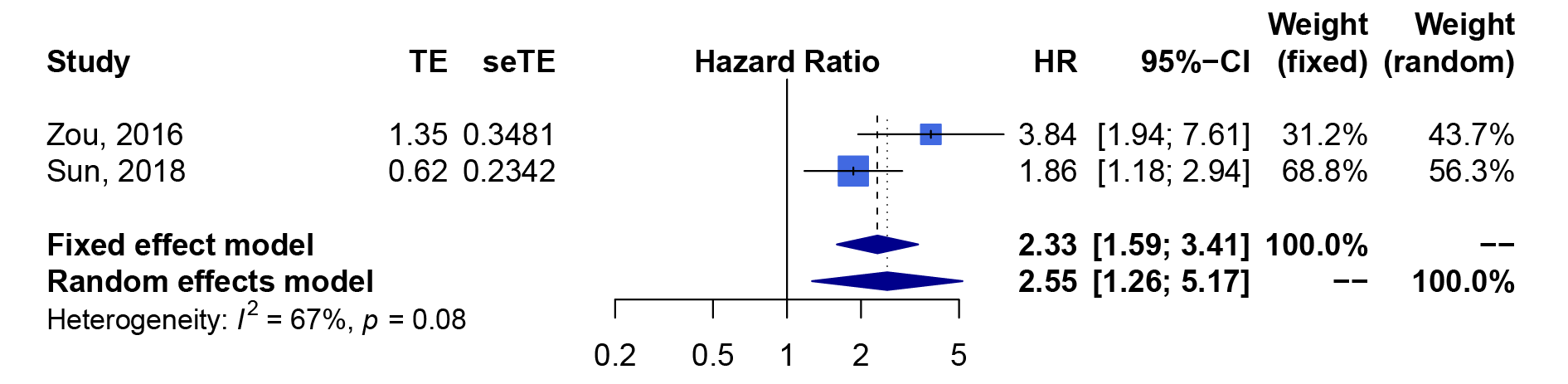

Supplement: Supplementary Figure 1 — Relationship of SOX2-OT expression and DFS in various cancers. [file Image_1.tif]

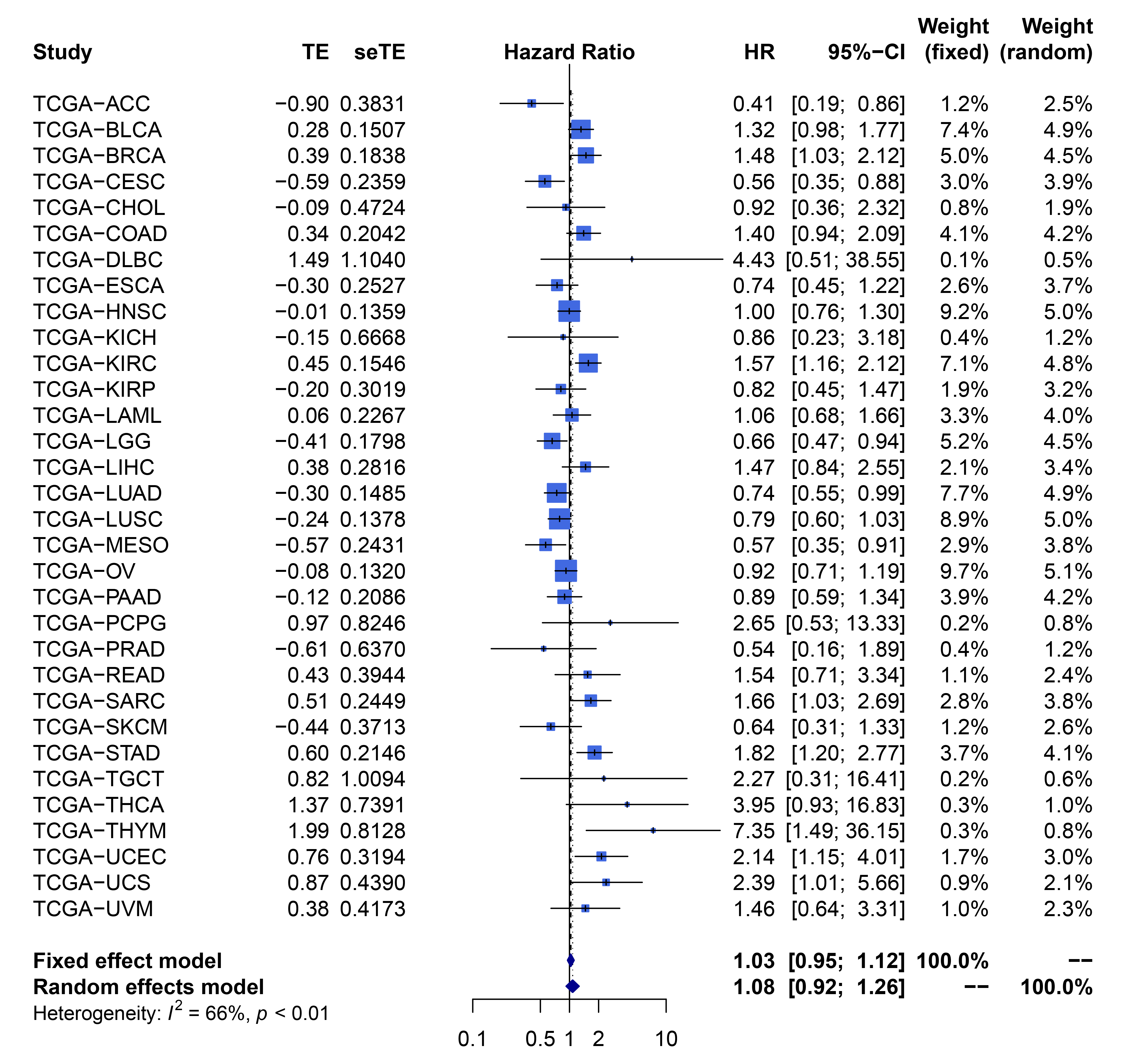

Supplement: Supplementary Figure 2 — Meta-analysis of TCGA datasets estimating the association of SOX2-OT with the patients’ overall survival (OS). [file Image_2.tif]

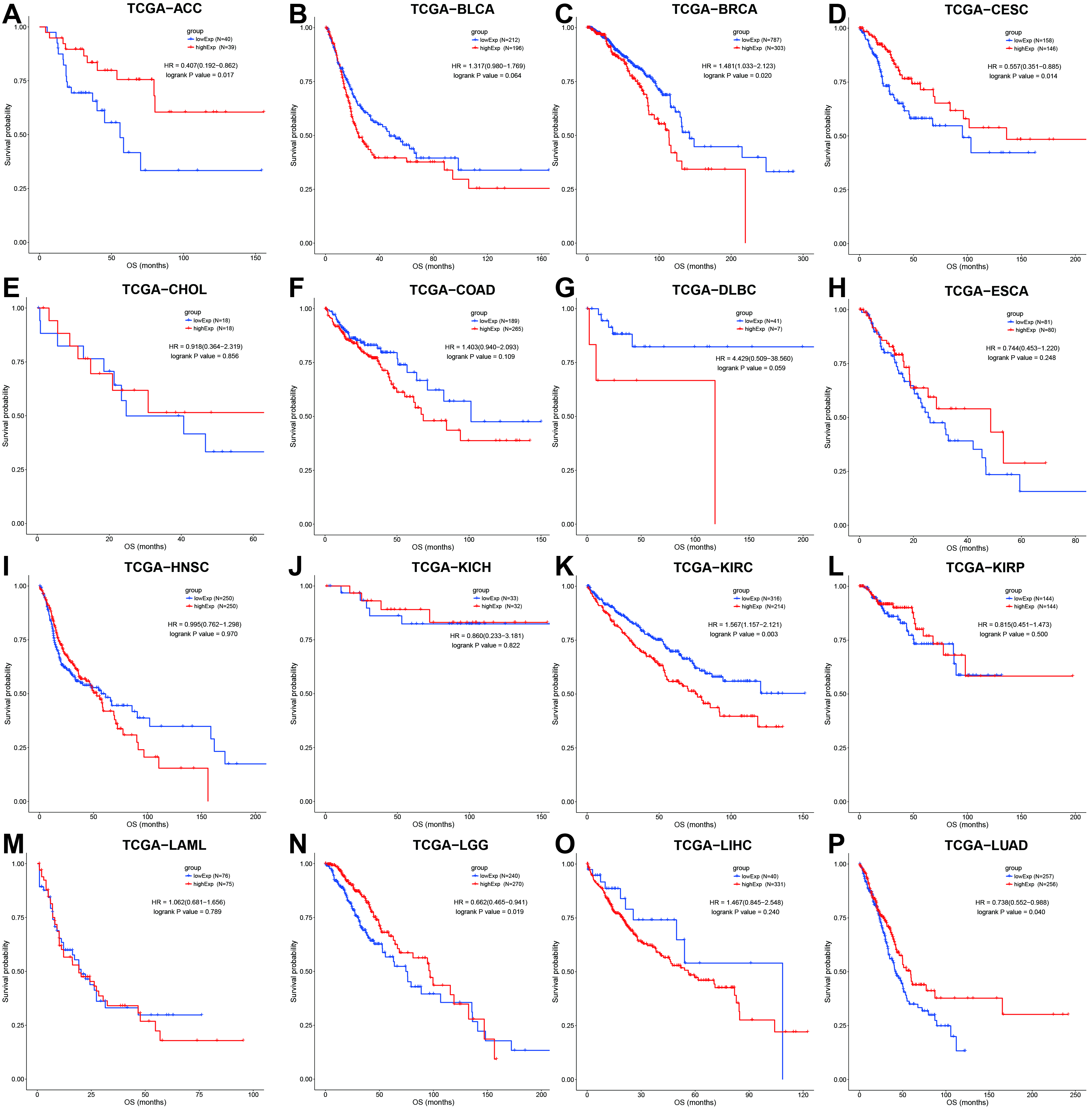

Supplement: Supplementary Figure 3 — Kaplan-Meier survival curves for overall survival of 16 cancers in TCGA, stratified by SOX2-OT expression levels. (A) ACC, (B) BLCA, (C) BRCA, (D) CESC, (E) CHOL, (F) COAD, (G) DLBC, (H) ESCA, (I) HNSC, (J) KICH, (K) KIRC, (L) KIRP, (M) LAML, (N) LGG, (O) LIHC, (P) LUAD. [file Image_3.tif]

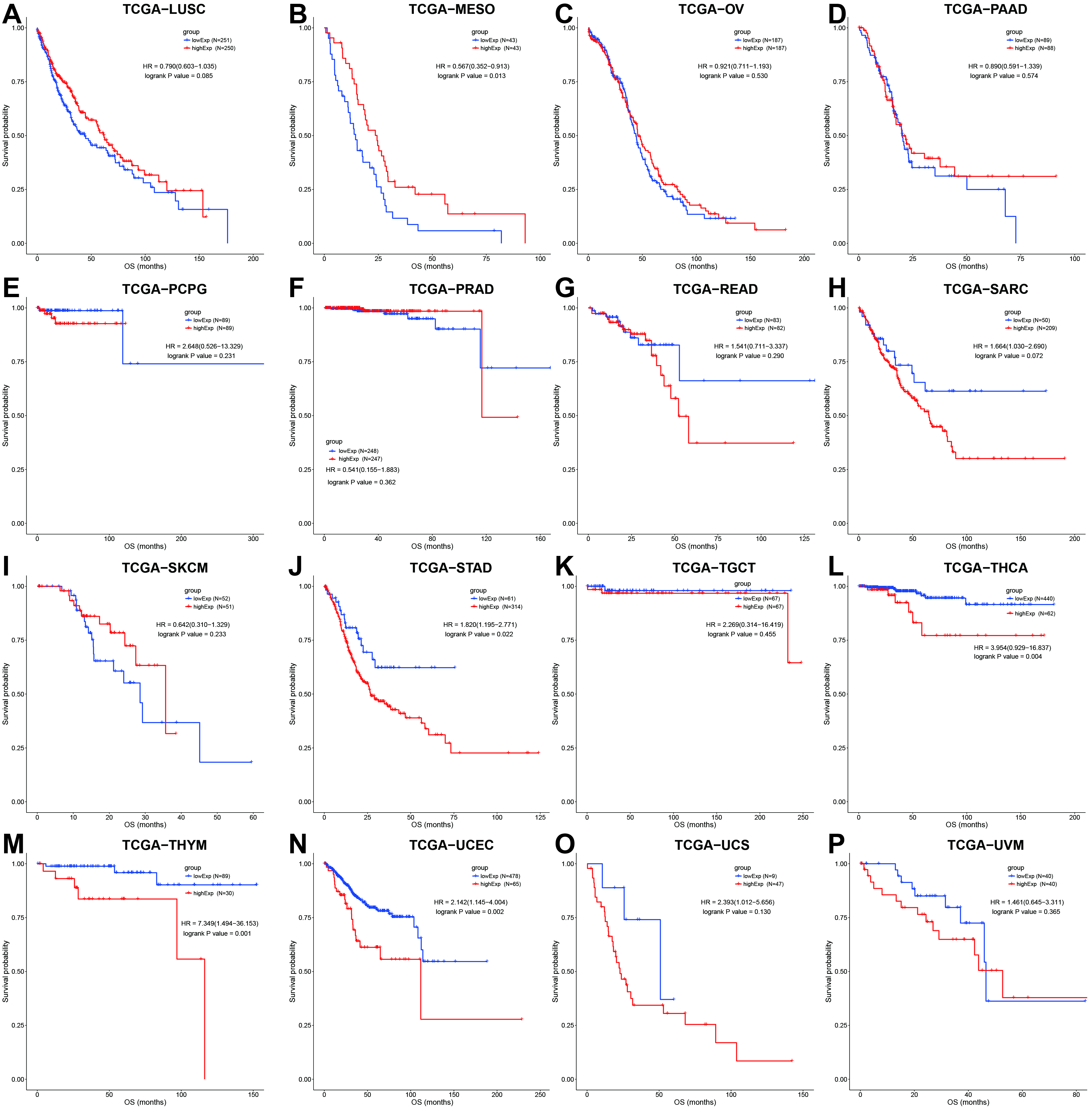

Supplement: Supplementary Figure 4 — Kaplan-Meier survival curves for overall survival of the other 16 cancers in TCGA, stratified by SOX2-OT expression levels. (A) LUSC, (B) MESO, (C) OV, (D) PAAD, (E) PCPG, (F) PRAD, (G) READ, (H) SARC, (I) SKCM, (J) STAD, (K) TGCT, (L) THCA, (M) THYM, (N) UCEC, (O) UCS, (P) UVM. [file Image_4.tif]

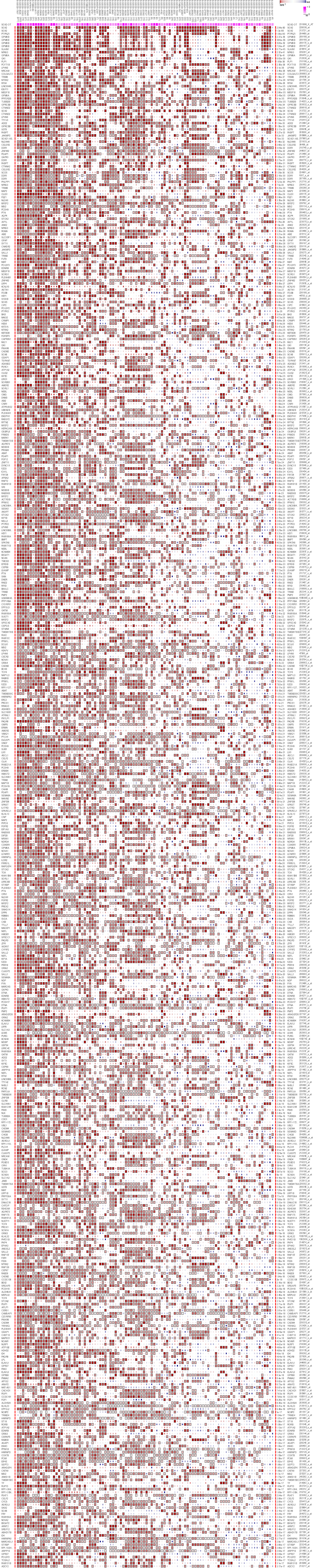

Supplement: Supplementary Figure 5 — Totally 500 target genes of SOX2-OT were identified with Multi Experiment Matrix (MEM). [file Image_5.tif]
