# Supplementary material for: Clinicopathological Implication of Long Non-Coding RNAs SOX2 Overlapping Transcript and Its Potential Target Gene Network in Various Cancers
Source: Front Genet. 2020 Jan 23;10:1375. doi: 10.3389/fgene.2019.01375 (PMC6989546; doi:10.3389/fgene.2019.01375)
Supplement: Supplementary Table 1 — Databases searching terms. (A) Search criterion of Medline (via PubMed, from inception to Jan 1st, 2019) (n=47), (B) Search criterion of Embase (from 1966 to Jan 20, 2019) (n=49), (C) Search criterion of Cochrane Library (Jan 1st, 2019) (n=1). [file Table_1.docx]

# Supplementary Table 1. Databases searching terms

**Supplementary Table 1A. Search criterion of Medline (via PubMed, from inception to Jan 1st, 2019) (n=47)**

| **Search**  **NO.** | **Query Results** | Items found |
| --- | --- | --- |
| **#1** | **SOX2-OT/ OR SOX2 overlapping transcript/ OR SOX2OT/ OR NCRNA00043/** | **66** |
| **#2** | **(((((((((((Carcinoma/) OR malig*[tw]) OR neoplas*[tw]) OR oncol*[tw]) OR tumor*[tw]) OR Neoplasms/ or adenocarcinom*[tw] cancer*[tw]))) OR tumour/ OR cancer/ or sarcom*[tw])))** | 4201546 |
| **#3** | **((SOX2-OT/ OR SOX2 overlapping transcript/ OR SOX2OT/ OR NCRNA00043/)) AND ((((((((((((Carcinoma/) OR malig*[tw]) OR neoplas*[tw]) OR oncol*[tw]) OR tumor*[tw]) OR Neoplasms/ or adenocarcinom*[tw] cancer*[tw]))) OR tumour/ OR cancer/ or sarcom*[tw]))))** | 47 |

##### Supplementary Table 1B. Search criterion of Embase (from 1966 to Jan 20, 2019) (n=49)

| **Search**  **NO.** | **Query Results** | **Items found** |
| --- | --- | --- |
| #18 | #12 AND #17 | 49 |
| #17 | #13 OR #14 OR #15 OR #16 | 72 |
| #16 | ncrna00043:ab,ti | 0 |
| #15 | 'sox2ot':ab,ti | 56 |
| #14 | 'sox2 overlapping transcript':ab,ti | 26 |
| #13 | 'sox2-ot' | 14 |
| #12 | #1 OR #2 OR #3 OR #4 OR #5 OR #6 OR #7 OR #8 OR #9 OR #10 OR #11 | 5517741 |
| #11 | sarcom*:ab,ti | 137707 |
| #10 | cancer:ab,ti | 2164462 |
| #9 | tumour:ab,ti | 305612 |
| #8 | cancer*:ab,ti | 2263150 |
| #7 | adenocarcinom*:ab,ti | 199809 |
| #6 | 'neoplasms'/exp | 4695931 |
| #5 | tumor*:ab,ti | 1815563 |
| #4 | oncol*:ab,ti | 248968 |
| #3 | neoplas*:ab,ti | 347572 |
| #2 | malig*:ab,ti | 783106 |
| #1 | 'carcinoma'/exp | 1199792 |

##### Supplementary Table 1C. Search criterion of Cochrane Library (Jan 1st, 2019) (n=1)

| **Search**  **NO.** | **Query Results** | **Items found** |
| --- | --- | --- |
| #1 | (SOX2 overlapping transcript) **:ti,ab,kw** | 0 |
| #2 | (SOX2OT) **:ti,ab,kw** | 1 |
| #3 | (NCRNA00043) **:ti,ab,kw** | 0 |
| #4 | #1 OR #2 OR #3 | 1 |
