# Supplementary material for: Clinicopathological Implication of Long Non-Coding RNAs SOX2 Overlapping Transcript and Its Potential Target Gene Network in Various Cancers
Source: Front Genet. 2020 Jan 23;10:1375. doi: 10.3389/fgene.2019.01375 (PMC6989546; doi:10.3389/fgene.2019.01375)
Supplement: Supplementary Table 2 — Results of quality assessment using the Newcastle–Ottawa Scale for the included studies. [file Table_2.docx]

**Supplementary Table 2.** Results of quality assessment using the Newcastle–Ottawa Scale for the included studies

| No. | Study | Selection |  |  |  | Comparability | Exposure |  |  | Scores |
| --- | --- | --- | --- | --- | --- | --- | --- | --- | --- | --- |
|  |  | Is the case definition adequate? | Represen-tativeness of the Cases | Selection of Controls | Definition of Controls | Comparability of Cases and Controls on the Basis of the Design or Analysis | Ascertainment of exposure | Same method of ascertainment for cases and controls | Non-Response rate | Total scores |
| 1 | Wang 2017 | ★ | ★ | ☆ | ★ | ★★ | ☆ | ★ | ★ | 7 |
| 2 | Zhang 2017 | ★ | ★ | ☆ | ★ | ★★ | ☆ | ★ | ★ | 7 |
| 3 | Han 2018 | ★ | ★ | ☆ | ★ | ★☆ | ☆ | ★ | ★ | 6 |
| 4 | Li ZL 2018 | ★ | ★ | ☆ | ★ | ★★ | ★ | ★ | ★ | 8 |
| 5 | Hou 2014 | ★ | ★ | ☆ | ★ | ★★ | ☆ | ★ | ★ | 7 |
| 6 | Shi 2015 | ★ | ★ | ☆ | ★ | ★★ | ☆ | ★ | ★ | 7 |
| 7 | Iranpour 2015 | ★ | ★ | ☆ | ★ | ★★ | ☆ | ★ | ★ | 7 |
| 8 | Zhang 2016 | ★ | ★ | ☆ | ★ | ★★ | ★ | ★ | ★ | 8 |
| 9 | Zou 2016 | ★ | ★ | ☆ | ★ | ★★ | ★ | ★ | ★ | 8 |
| 10 | Xie 2018 | ★ | ★ | ☆ | ★ | ★★ | ☆ | ★ | ★ | 7 |
| 11 | Sun 2018 | ★ | ★ | ☆ | ★ | ★★ | ☆ | ★ | ★ | 7 |
| 12 | Li ZH 2018 | ★ | ★ | ★ | ★ | ★★ | ☆ | ★ | ★ | 8 |
| 13 | Wei 2018 | ★ | ★ | ☆ | ★ | ★★ | ☆ | ★ | ★ | 7 |
